# Public Involvement in Complex Theorising: A Co‐Produced Logic Model of the Role of Context in Shaping Child Health

**DOI:** 10.1111/hex.70346

**Published:** 2025-08-04

**Authors:** Dylan Kneale, Alison O'Mara‐Eves, Bridget Candy, Lizzie Cain, Jessica Catchpole, Angela Chesworth, Sandy Oliver, Katy Sutcliffe, Niccola Hutchinson Pascal, James Thomas

**Affiliations:** ^1^ EPPI Centre, Social Research Institute University College London London UK; ^2^ Marie Curie Palliative Care Research Department, UCL Division of Psychiatry University College London London UK; ^3^ Innovation Unit London UK; ^4^ Department of Nursing, School of Health & Psychological Sciences, City St George's University of London London UK; ^5^ Independent Patient Advocate UK; ^6^ Co‐production Collective University College London London UK

**Keywords:** children's health, co‐production, complex systems, logic model, weight management

## Abstract

**Introduction:**

There is increasing work towards drawing on theory, implementing co‐production and accounting for complexity within the production of systematic reviews for public health. In this paper, we report on the process of co‐producing a theory; in this case, a graphical articulation of theory in the form of a logic model, which describes how contextual factors influence children's health.

**Methods:**

We undertook a series of three online co‐production workshops, involving 18–20 participants in each, and worked with an advisory group of experts with professional and lived expertise. An online virtual whiteboard was used to support the identification of factors that contributed to poorer childhood health, explanations for these factors, and connections between different factors.

**Results:**

Driven by government strategy, we initially focussed our work on childhood obesity. However, co‐production was transformational in switching the focus of the logic model away from a narrow focus on Body Mass Index as a measure of obesity, to a more holistic theory of factors that shape children's health, recognised as the intersection between healthy eating, physical activity and mental well‐being. Theorising with a diverse range of co‐producers helped us to recognise the stigmatising impacts that an exclusive focus on clinical measures of children's health can have, and the way that a narrow clinical focus inhibits theorising the complexity and drivers of poorer health.

**Conclusion:**

Co‐production led to a switch in theorising away from narratives of children's health that focus closely on personal responsibility, towards narratives that explore structural and contextual drivers of health.

**Patient or Public Contribution:**

The logic model was entirely driven by the contributions of researchers, those with lived experience (e.g., as parents and/or who have experienced poor health), and those with professional experience (e.g., as teachers) who worked together to co‐produce the model. An advisory group composed of people with a similar range of expertise helped to shape the conduct of co‐production and dissemination (including in the preparation of this manuscript).

## 1 Introduction

Theory is essential in all research, and the role of theory is increasingly recognised in evidence synthesis [1, 2]. Theories—whether implicit or explicit [2], grand, middle or low level [1]—are a product of synthesising knowledge and involve articulating different concepts and their relationships, allowing generalisation beyond immediate scenarios [2]. However, different forms of knowledge are valued unequally and, historically, knowledge gained through lived experience of a health condition or service has been undervalued in theory development [3]. In this study, we report on the process and results of research where a theory around the influencers on children's health was co‐produced in the form of a logic model.

Logic models are a graphical approach to visualising and/or creating theories around how an intervention activates a series of processes that are thought to create change [4]. A broad distinction can be made between process‐based logic models, which support theorising of the granular steps taking place within an intervention or service, and systems‐based logic models. The latter involves greater theorising of the relationship between an intervention and its context than the former [5, 6].

Alongside a trend towards clearer articulation of theory (and particularly logic models [7]), two other trends are directing evidence synthesis methodology.

The first of these is a recognition of the value of public and patient involvement (PPI) in the creation of evidence syntheses [8]. In particular, co‐production is recognised as a way of transforming the relationship and power differentials between researchers and non‐researchers to enable more equitable decision‐making [3, 9]. Co‐production can be hugely beneficial to the quality of research, although these benefits are challenging to quantify [3, 10], and the literature points towards an array of benefits and challenges of co‐producing research, some of which are presented in Box 1.

Box 1 **Possible benefits and challenges of co‐producing research that have been proposed.**

**Table:** 

Possible benefits of co‐producing research	Possible challenges of co‐producing research
Promotes epistemic equity by valuing and integrating knowledge from lived experiences [9, 11].	Managing the expectations of co‐producers regarding the potential of research projects to address their issues [12, 13].
Contributes to decolonising knowledge production systems [14].	The potential for co‐production to place responsibility on co‐producers to resolve problems they cannot solve [12, 13].
Integrates different perspectives and synthesises transdisciplinary knowledge, making research more useful and usable [15].	Whether issues of representation and power in research are adequately addressed or not [10, 16].
Promotes inclusion, especially with historically marginalised groups [3, 15].	The practicality of added resources needed to undertake meaningful co‐production [10, 17].
Enhances trust between communities and academic institutions.	The wide spectrum of approaches that identify as co‐produced research, leading to variability in implementation [3, 15, 16].
Increases the legitimacy of research.	Challenges arising from abandoning or failing to enact core principles of co‐production [9, 18].
Aligns with a complex systems perspective, accommodating emergent outcomes and non‐linear processes [9].

The third trend is a movement towards recognising and unpacking the role of complexity within evidence syntheses, and particularly the wider contexts within which interventions are implemented [19]. However, understanding which specific features of context may influence health outcomes is often not fully understood and is under‐theorised [20].

In this study, we integrated these three elements (logic models, PPI and unpacking complexity) within a project that sought to co‐develop a theory (a systems‐based logic model) around children's health (and initially, specifically around obesity) that was focussed on identifying which contextual features harmed or promoted children's health.

### 1.1 Childhood Obesity and Theorising Factors That Drive Childhood Obesity

Childhood obesity has multiple health implications [21]. The global prevalence of obesity has increased over the last decades and is set to continue [22]. Theoretical frameworks to understand the causal mechanisms and context of childhood obesity have historically focussed on individual behaviour change, such as the Theory of Planned Behaviour [23]. Alongside recognition of the broad context of influences on obesity, whole‐system approaches are gaining greater traction. Such approaches in the context of obesity are a way of considering how individuals, groups, services and organisations interconnect and influence each other [23]. They allow consideration of both immediate and more distal contextual factors, including, for example, industry and government policy. These approaches support conceptualising the drivers of obesity as a complex web of interacting relationships that give rise to what is observed as obesity. One such whole system approach is the UK government's Foresight Obesity report [24], a key output of which was a map of drivers theorised to influence (adult) obesity, which is seen as a turning point where obesity came to be understood in terms of whole systems complexity [25].

The Foresight Obesity Systems Map visually sets out the complexity of the drivers of obesity, which fall within the responsibility of multiple agents (e.g., individual, commercial, voluntary sectors and government) [26, 27]. There are 108 drivers of obesity included in the Foresight Systems map, which are divided into seven interlinking areas of influence on a person's energy intake: individual biology, individual activity, environmental activity, individual psychology, societal influences, food consumption and food production. All these link to an individual's energy balance, placed in the centre of the system. What visually dominates the systems map is the over 300 lines that connect the factors, alongside feedback causal loops of different sizes, dependent on the likely strength of influence.

The Foresight model is of interest here as it takes a systems perspective and provides a theory of influencers on obesity. However, it was based on the input of scientific experts, who were asked what they believed to be important and who collectively published 34 reviews and opinion pieces that contributed to the development of the model [28]. ‘Non‐scientific’ input into the development of the Foresight model included representatives from government departments, as well as the retailer Tesco, among others [25]. Crucially, the development of the model did not involve people with lived experience. Moreover, it was not specific to the initial focus of this study—examining childhood obesity—which plausibly has very different factors and pathways to a general or adult‐focussed model.

## 2 Methods

### 2.1 Study Aims

The aims of this study were to:

i. develop a systems‐based logic model of the factors that influence children's capacity to maintain a healthy weight and
ii. to understand what happens when a logic model is co‐produced through the input of a range of experts.

The logic model was to focus on the context surrounding interventions that are implemented in schools. The logic model was to be used to inform a larger project on evidence synthesis methods (not reported here) that involved the development of novel meta‐analysis methods. These new meta‐analytic methods were intended to support the exploration of context within systematic review evidence, but were not co‐produced. However, the selection of which contextual factors to explore in the meta‐analytic methods was intended to be grounded in theory (i.e., derived from the co‐produced systems‐based logic model).

In this paper, we provide an example of the value of assembling a diverse co‐production team to co‐produce a logic model through:

i. Providing an account of how co‐production facilitated a shift in the model's focus.
ii. Presenting the key features of the resulting logic model (with access links), which can support further research into the contextual factors that shape children's health and influence the implementation and effectiveness of school‐based interventions.

### 2.2 Setting and Context

During the midst of the pandemic in 2020, and with evidence showing that obesity was linked with a worse Covid‐19 prognosis [29], the government launched a new strategy for child and adult obesity [30]. This was the fourteenth obesity strategy launched in the past three decades [31]. While the strategy was a marked departure from several previous strategies in containing a greater number of policies that sought to change the availability of unhealthy foods (e.g., banning price promotions around unhealthy products) [31], not all of these policies were actually implemented [32]. Notably, the strategy did not include policies targeted solely at physical activity [31], but did contain policies targeting diet alone, or diet in conjunction with physical activity.

The project took place between 2021 and 2022, when Covid‐19 pandemic restrictions on social gatherings were still in place for part of this study. All of the work took place remotely, with the academic research team neither meeting with one another nor with new co‐producing colleagues. The whole project—co‐production of a logic model and development of meta‐analytic methods—was conducted within a rapid time frame of 10 months.

### 2.3 The Academic Research Team and AG

The UCL‐based research team brought a range of experiences, including in evidence synthesis, public health, children's participation and rights, weight management research and experiences, and around participatory methods (although this had been more in involvement and engagement than co‐production). To support co‐production, we worked in partnership with members of the Co‐Production Collective, who were instrumental in training us on the values, methods and processes of co‐production (see [33]).

To support the research, we assembled an Advisory Group (AG) of experts with lived and professional experience. The AG provided methodological advice on co‐production through lived experience and provided support for other parts of the larger project, including with advice on meta‐analytic methods. We approached membership and management of this AG through the principles of co‐production, although the AG was not directly involved in co‐producing the logic model. These principles include: (i) challenging the status quo through ongoing critical reflection as a group; (ii) working in accessible and inclusive ways; and (iii) embracing diversity in perspectives and knowledge (through bringing together lived and professional experiences in the membership of the group).

In total, nine people agreed to join the AG, including academics with specialisms in evidence synthesis and health inequalities (3; 2 did not attend meetings), public health practitioners (2), school teachers (1) and people with lived experience (2). The AG was given email updates and opportunities to comment throughout the project, and met (virtually) three times. All meetings were hosted on Zoom (see Table 1 for the schedule of meetings).

**Table 1:** Summary of Advisory Group schedule.

Meeting number	Schedule and contents
1	Involved informing the group of the purpose of the study, providing an opportunity for members to get to know each other, clarifying expectations for the research and determining how to organise the co‐production of the logic model.
2	Occurred after the stages of co‐production to discuss the draft logic model and its implications for future work.
3	Occurred after a working draft of the model had been developed, and when we had progressed to using the model to inform the development of meta‐analytic methods.
4	Planned, but it was deemed that final inputs would be best gained through email contact. This was in part due to the time frame of the work, which was already tight, but was further compounded by the emergence of a new coronavirus variant, placing strain on people's availability.

One crucial way the AG strengthened the research was by advising the UCL‐based team on how to have conversations about sensitive topics like obesity when co‐producing the logic model. Unfortunately, scheduling conflicts meant that the full AG was not able to convene at all times. However, those with lived experience attended all meetings, which had the unexpected advantage of balancing the numbers—and possibly the power dynamics—between contributors with lived experience and those engaging through professional experience. While the AG members were intended to occupy a role that was exterior to the co‐production team, UCL‐based co‐producers came to regard them, particularly both AG members with lived experience, as integral to the project and as co‐producers and co‐authors of this article.

### 2.4 Forming a Team for Co‐Producing a Systems‐Based Logic Model

To co‐produce a systems‐based logic model, we planned a series of online workshops. The co‐production workshops were designed to gain insights into contextual factors relating to child health. Two initial workshops were held to develop the model, followed by a third workshop with a broader audience to check and challenge it. The workshops were conducted virtually and hosted on Zoom and were scheduled to last 2 hours.

#### 2.4.1 Co‐Production Workshops 1 and 2

##### 2.4.1.1 Focus

The first workshop was intended to identify important concepts, and the second workshop was intended to identify the relationships between these concepts.

##### 2.4.1.2 Recruitment

Recruitment of co‐producers based outside UCL took place informally through a mixture of targeted and open recruitment. Targeted recruitment drew on the personal and professional links of the academic research team and the Co‐production Collective. Open recruitment was through mailouts, blogs hosted on the Co‐production Collective website, and a website for the project. Remuneration for people's time was offered based on guidance and included attendance at the workshops plus preparation and/or follow‐up work [34].

The main area of expertise of co‐producers included in Workshops 1 and 2 is presented in Table 2 (although co‐producers may have had multiple areas of expertise). A member of Co‐Production Collective facilitated workshops and liaised with co‐producers before and after the workshops. All co‐producers were aged 18+, a limitation that we reflect upon in our discussion.

**Table 2:** Co‐producer main area of relevant expertise across co‐production Workshops 1 and 2.

Co‐producer main area of relevant expertise	Workshop 1	Workshop 2
UCL‐based co‐producers (public health evidence synthesis specialists)	5	5
Co‐Production Collective	2	2
Lived experience	4	5
GP and advocacy	1	1
Teaching (school teachers)	4	3
Occupational therapy	0	1
Academic research (public health)	2	2
Nutrition and advocacy	0	1
Total	18	20

##### 2.4.1.3 Process

**Figure 1:**
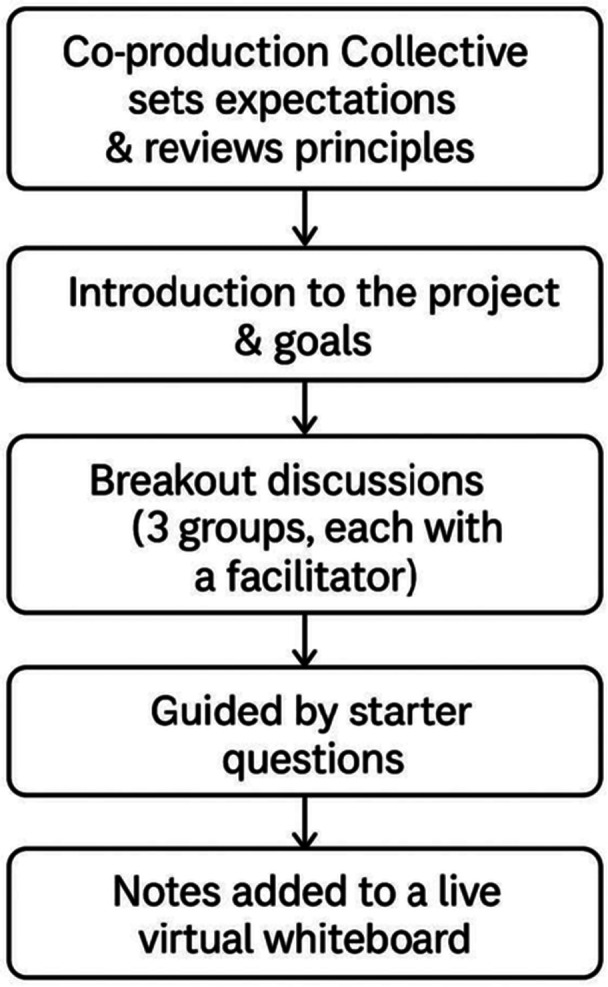
Process for Workshops 1 and 2.

The first two workshops followed a similar pattern and are summarised in Figure 1.

To facilitate the discussion in Workshop 1, we drew on the ‘domains’ identified in the Obesity Foresight map and expanded on these, and asked people to consider factors relating to the following domains: (i) food; (ii) biological factors (explained as things to do with how the body works including genetics and metabolism); (iii) social factors (which could be personal, community and cultural factors); (iv) activity and behavioural factors; (v) developmental factors (things to do with age transitions); (vi) economic factors (things to do with spending and earning money); (vii) infrastructure and environmental factors (things around us in our daily lives such as transport and buildings); (viii) psychological factors; and (ix) media. We also added (x) medical factors (things to do with illness and injuries, including the side effects of medications); (xi) school factors (what happens in school); and (xii) a space for all co‐producers to identify ‘other’ factors that did not fit into the other categories.

UCL‐based co‐producers shared with the team that much of what might be theorised within the model might not be captured within studies, as people's experiences are often more complex than represented in the literature. However, the UCL‐based co‐producers also emphasised that this did not belie or detract from the importance of theorising these factors. This was part of efforts to be clear about the expectations of how the model was going to be used initially within the project.

The first two workshops were spaced 3 weeks apart to keep the momentum. Between the first and second workshops, two of the UCL‐based co‐producers (D.K. and A.O.M.E.) attempted to group the virtual post‐it notes that had been recorded and consolidate any duplicates. In grouping the notes, it was also identified that the factors represented on the post‐its also fell within different ‘ecological’ levels of influence. We used an adapted social‐ecological model [3] as the basis for organising the notes subsequently. The post‐its were grouped across the following levels: (i) individual; (ii) household, family and friends; (iii) school; (iv) neighbourhood (place‐based); (v) cultural community (incl. social media); (vi) economic systems; (vii) socio‐political, infrastructure, national policy and media; and (viii) cross‐cutting factors (temporal and historical trends).

The arrangement of the different factors identified in Workshop 1, the configuration of the outcome, and the arrangement of factors across the different levels of influence became the main sources of discussion in Workshop 2.

#### 2.4.2 Co‐Production Workshop 3

##### 2.4.2.1 Focus

Once the themes/concepts were organised, we needed a visualisation method that: preserved the levels and concepts; could represent factors and more granular subfactors; could represent ‘logical’ relationships; and could preserve the original sentiments for reference. We continued to use Miro for this visual representation. The aim of Workshop 3 was to check and challenge the logic model and to introduce new methodological developments.

##### 2.4.2.2 Recruitment

We held a third workshop, which involved co‐producers, plus a new group of policymakers and practitioners. Workshop 3 had 12 attendees based outside UCL: 7 ‘returners’ and 5 new attendees recruited through our networks. While all co‐producers were invited to all three workshops, scheduling conflicts, as well as a relatively long interval (2 months) between Workshops 2 and 3, meant that there was substantial attrition in the number of ‘returners’.

##### 2.4.2.3 Process

A link to the interactive model and a short instructional video were provided before the workshop. During the 2‐h workshop, UCL‐based co‐producers presented the project progress and then used breakout groups to seek feedback from the co‐production team and the wider set of participants.

None of the co‐production workshops were recorded to enable co‐producers to speak more freely about their experiences and to voice their opinions.

### 2.5 Study Data

The results reported here are based on:

1. An analysis of the features of the co‐produced logic model.
2. Summaries of informal reflective discussions among UCL‐based co‐producers that took place after co‐production workshops and within AG meetings.
3. Summaries of reflections submitted by co‐producers based outside UCL, who were invited to submit their reflections to the Co‐Production Collective. These were summarised by LC.

These helped us to identify the distinctive contribution that co‐production made to the development of the logic model.

### 2.6 Ethics

This study followed the Economic and Social Research Council's research ethics framework. Ethical approval was gained from the UCL's Institute of Education (REC 1498). In line with institutional policy at the time, informed consent was sought from co‐producers based outside UCL for participation in the workshops, although we acknowledge that obtaining consent is not usually necessary in cases where co‐producers are equal partners in the research.

## 3 Results

The aims of the study were to develop a systems‐based logic model of the factors that influence children's capacity to maintain a healthy weight and to understand the influence of co‐production. The following sections describe the product of this study where we highlight key points in which the unique contributions of the collaborators from beyond the core research team were particularly evident in developing the logic model.

### 3.1 The Switch in Focus From Childhood Obesity to Children's Health

Much of the discussion in the first workshop centred around the outcome that should be the central focus of the logic model. The UCL‐based co‐producers had used childhood obesity as a starting point, and based on previous experience conducting reviews in this area, had anticipated that measures of weight (and particularly Body Mass Index [BMI]) would predominate as the primary outcome in the intervention literature. The broader co‐production team was unanimous in questioning the value of the focus solely on children's BMI. Co‐producers with lived experience of obesity or childhood obesity and parents voiced concerns that obesity was a hugely stigmatising condition for children and that this stigma was as damaging to the transition to adulthood as the health implications of obesity—and likely much more so. A particular concern to one co‐producer was the ‘malleability’ of BMI to the influence of public health interventions. Among health practitioners in the group, it was felt that standardised measures of weight, such as BMI, could be useful in decision‐making, although they had their limitations. For public health practitioners and researchers, BMI as a measure was recognised as a narrow construct and was not a useful reflection of the complexity of children's health. Further, there was no support for energy balance as a primary outcome of the model, despite this being the central focus of the original Foresight Obesity model.

**Figure 2:**
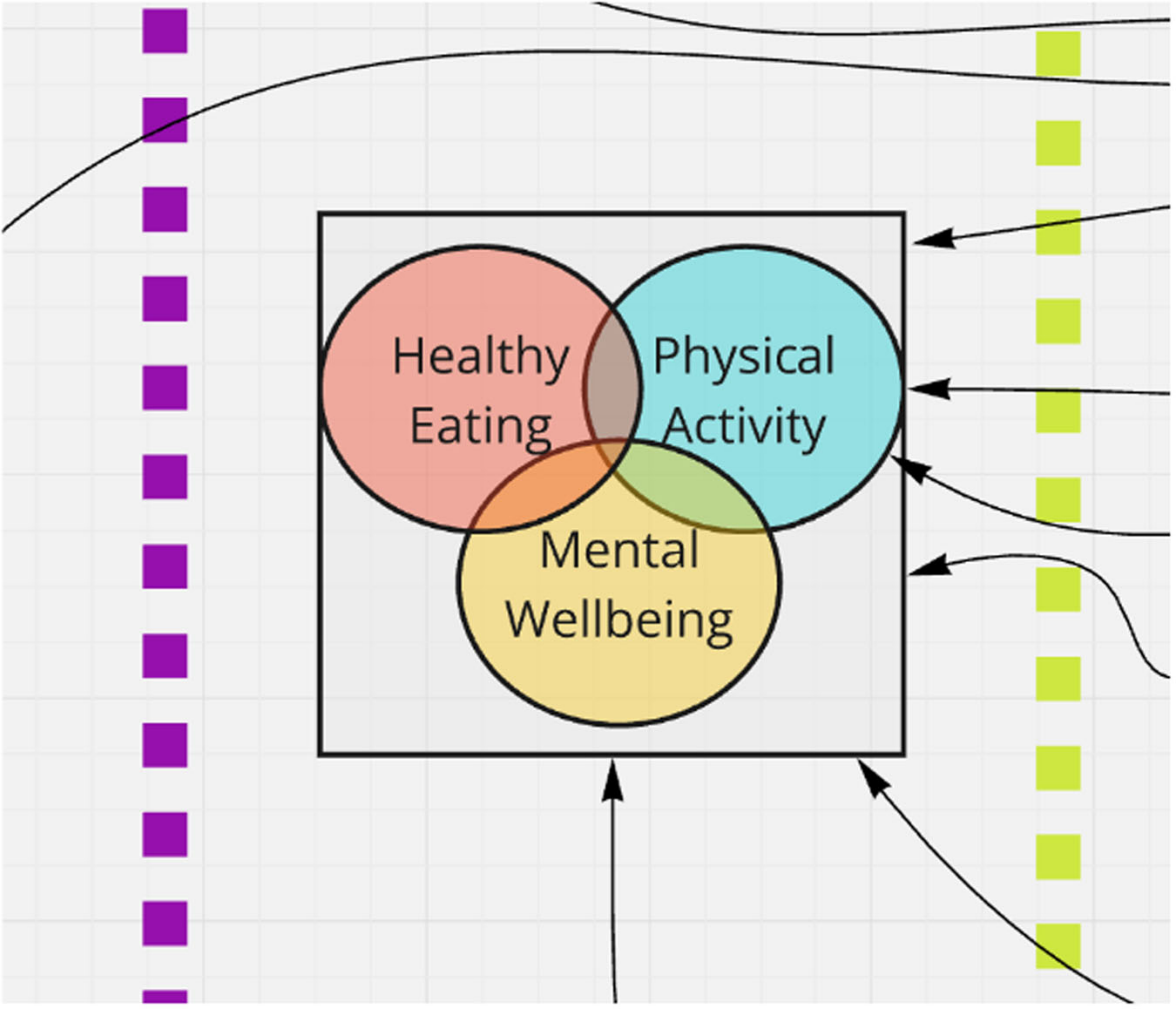
Section of the logic model (see explanation in text).

During the first co‐production workshop, it was agreed that the outcome central to the logic model should reflect a broader conceptualisation of children's health than BMI. Much of the discussion, reflecting the expertise in the room and the framework used, continued to focus on influences on children's diet and physical activity, as well as factors influencing childhood mental health. Between the first and second workshops, the UCL‐based co‐producers aimed to seek clarification around the parameters of the broader outcome and proposed in the second workshop that children's health should be operationalised in the model as the intersection between healthy eating, physical activity and mental health (see Figure 2). While diet and physical activity are often co‐occurring as the foci of multicomponent weight management interventions [35], mental health is often treated only as a secondary outcome or neglected altogether. In addition, healthy eating and physical activity (HEPA) were already recognised as co‐occurring in some strategies; the addition of mental well‐being (creating the acronym HEPAM) was viewed as reflecting the concerns of all co‐producers that issues around stigma and weight were underappreciated within the debate within this space.

The focus on HEPAM—where good child health is determined through the intersection of healthy eating, physical activity and mental well‐being—was viewed by almost all co‐producers as a satisfactory way of attempting to capture the complexity of the discussions within the first workshop. They were viewed as joint outcomes that school‐based interventions could theoretically address. However, for some co‐producers, the switch in outcome from initial proposals to examine BMI to HEPAM was not transformational enough in addressing concerns around the damaging impacts of labelling children as obese or, likewise, as ‘unhealthy’.

### 3.2 An Overview of a Systems‐Based Logic Model of Children's Health

The central outcome of the logic model is a broad conceptualisation of children's health that reflects an intersection between healthy eating, [partaking in] physical activity and mental well‐being. The model is organised according to two central principles—that influencers on children's health can be grouped into broad domains (i.e., different types of factors) and that influencers on children's health occur at different socio‐ecological levels (Table 3). Due to its complexity, the model is not reproduced here, although the link to the model is available here, and a link to a video explaining how the model can be accessed is available here (zoom account required).

**Table 3:** A description of the contents of the different domains within the logic model (see text for further information).

Domain name	Example strand	Number of factors	Number of subfactors	Number of levels in which the domain is represented
Psychological	Children's agency <SF> Household dynamics ‐> Children's psychology ‐> Motivation for Healthy Behaviours ‐> HEPAM	14	11	5
Infrastructure and environment	Access to leisure resources ‐> Children's environment ‐> Capability to be healthy ‐> How children spend their time ‐> HEPAM	4	3	3
Media	Social media ‐> Motivation for healthy behaviours ‐> HEPAM	3	2	1
Biological and medical	Interaction between genes and environment <SF> Genetics ‐> HEPAM	3	1	1
Activity	Screen time <SF>How children spend their time ‐> HEPAM	8	6	6
Schools	Policies around how children spend their time ‐> How children spend their time within school ‐> HEPAM	6	6	2
Developmental	Puberty ‐> HEPAM	2	1	1
Economic	Globalisation <SF> Macroeconomic policy and values ‐> Poverty ‐> HEPAM	9	4	3
Food	Household attitudes to food <SF> Child attitudes to food ‐> [Capacity to make] Informed choices ‐> What we eat and drink ‐> HEPAM	15	24	7
Other	Evidence ecosystem (how evidence is used)	7	15	3
	Total	71	73	8 (including cross‐cutting trends)

Abbreviations: ‐> = Influences, <SF> = Subfactor/feature of.

**Figure 3:**
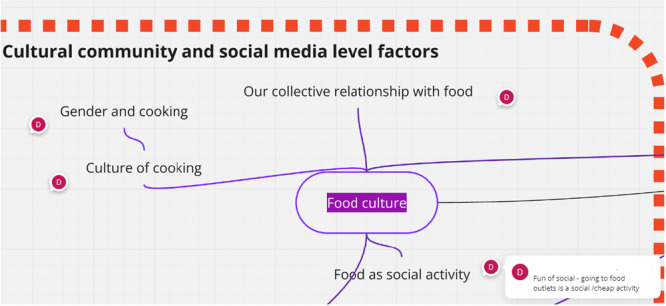
Section of logic model focusing on food culture themes (see explanation in text).

Figure 3 shows a snapshot of a small sub‐section of the model, which focuses on cultural community factors and a section of food as a domain. Here, we can see the factor (food culture) and a number of subfactors relating to food culture. Each circle with the letter ‘D’ indicates that further text providing an explanation is available; in the example below, the social side of food and the reality that going to food outlets is a social/cheap activity is used to support ‘food as a social activity’. The model here recognises that school‐based interventions to improve children's health may be implemented in contexts where eating out is a social activity of significance. These descriptions provide an approximation of why we, as a co‐production team, determined the factor to be an important influencer on children's health in the context of school‐based interventions.

Factors influencing children's health were organised according to broad domains (see ‘Methods’). These domains are colour‐coded, and more granular models that show both the factors (core concepts) and subfactors (granular details) belonging to a domain were also created as separate models (see Table 4 for an overview).

**Table 4:** Discussion points presented to co‐producers across co‐production Workshops 1 and 2.

Discussion points for co‐producers—Workshop 1
A first question to consider as a group—what should we have as our central concept? ▪ Our preferred concept is weight ▪ Other concepts we could/did consider healthy weight, obesity/overweight (public health focus) ▪ Another concept we could consider is energy balance (clinical focus) Some further questions for breakout groups ▪ What are some of the community influencers on children's weight? ▪ What are some of the things that happen in school that can influence children's weight? We want to make sure ideas are supported by examples/ideas as much as possible (e.g., this factor is important because….)

Discussion points for co‐producers—Workshop 2
Building on the inputs from Workshop 1, we asked co‐producers for: Looking at the factors grouped into themes on the grid: ▪ Are any factors missing? ▪ Do any factors belong elsewhere? Each group to look at 2–3 levels (school – > outer level) of the model and discuss: ▪ What we think the levels mean? ▪ Do we agree with the factors included at each level? ▪ Does looking at different levels help to think about different factors? ▪ Do any factors occur at more than one level?

These factors can also occur at different points in children's lives. For example, ‘how children spend their time’ is a factor that is influenced by, and occurs within, schools, neighbourhoods, culture, economic systems, households and families, and by factors occurring at the individual level. A number of the levels and factors are clearly beyond the purview of any individual policy, service or intervention to improve children's health (e.g., a school‐based intervention to improve children's health could not influence broad culture). They may, however, be important factors to consider in interpreting evidence, and particularly in interpreting results across different settings (as is often the case in systematic reviews).

### 3.3 Personal Choice vs. Upstream Determinants

A noteworthy feature of the model is its focus on upstream determinants. Co‐producers based outside UCL questioned the extent to which individual factors reflected genuine ‘choices’ when individuals were making those choices in the shadow of large companies and inadequate policy responses. For example, some of us in the co‐production team described how the ‘food industry working actively against the driver—promoting idea of individual choice/blame’ and questioned ‘morals in food industry’. In this respect, the model reflects the ‘commercial determinants of health’ that others have also cited as disproportionately responsible for poor public health outcomes [35]. Such commercial determinants may be particularly important to consider in the case of children, who may have less control over their environments.

The model also includes a concern around the commercial determinants of health and the role that online media plays in shaping children's health. In fact, within the media domain, there was little mention of factors relating to broadcast media (TV or online streaming) and more concern around social media and social networking as determinants of HEPAM, and the model includes no mention of print media.

In line with discussions around the broad conceptualisation of children's health, there was concern that there had been a disproportionate focus in society and healthcare policy on individual and parental responsibility. A factor directly reflecting this around ‘the [misplaced] onus on individual not societal responsibility’ was included. Table 5 also shows that under a third of factors and subfactors named were placed in the individual or household, family and peer networks levels (29% [43/146]) with a far greater number placed in levels reflecting socio‐political, economic and cultural and community levels (42% [61/146]). Co‐producers based outside UCL also critiqued the language of policy around obesity, which routinely describes children being in the midst of a ‘war’ or ‘crisis’ or ‘epidemic’.

However, despite being more orientated towards acknowledging commercial, social and political determinants of children's health, some participants at the end of the third workshop indicated that environmental factors had still been downplayed, that mental health did not occupy a sufficiently prominent position, and that government choices such as austerity were not adequately represented.

**Table 5:** Number of factors related to each domain (psychological, media, etc.) represented in the different levels (individual, socio‐political, etc.) within the logic model (see text for further information).

	Domain										
	Psychological	Infrastructure and environment	Media	Biological and medical	Activity	Schools	Developmental	Economic	Food	Other	Total
Socio‐political	3	0	0	0	0	2	0	4	5	12	26
Cultural community and social media	4	0	5	0	2	0	0	0	5	2	18
Economic	0	2	0	0	2	0	0	8	5	0	17
Neighbourhood	0	3	0	0	1	0	0	0	3	0	7
School	0	0	0	0	1	10	0	1	6	0	18
Household	3	0	0	0	2	0	0	0	7	0	12
Individual	11	0	0	4	6	0	2	0	8	0	31
Cross‐cutting trends	4	2	0	0	0	0	0	3	0	8	17

### 3.4 What the Model Does Not Cover

As noted in the ‘Methods’, the first workshop was intended to identify different concepts and the second workshop was intended to identify the relationships between different concepts, but the second ambition was not fully addressed. We ran out of time in the first workshop, in part reflective of budgetary and administrative constraints, and collectively agreed that it would be more fruitful to ensure that we had good coverage of key factors rather than moving on to look at relationships between an incomplete set of factors.

As a result, we have very few posited pathways between factors. Those that are included were explicitly discussed in workshops; there are likely to be others. In addition, the model does not tell us about: the strength (magnitude) of any effects; mediating or moderating factors; or whether any factors are supported by empirical evidence. While the methods used in this study were not intended to develop an empirically evidenced model, future research could build on our theorised system and test the elements and putative pathways within the model.

Finally, as a part of a system, it is acknowledged that changes to any one factor in the system may lead to changes or adjustments in others, although the model does not make predictions about how any reactions or substantial change within the system might occur.

## 4 Reflections

Among co‐producers from outside UCL, who brought a diversity of experiences (see Table 1), the change of focus from child obesity to child health, and evidence that their input was recognised, was highly valued. Many reported feeling that they personally benefitted from participating in the project. Some indicated that they are very keen to take part in further research, and some participating as researchers (outside UCL) had heard perspectives around weight‐based stigma which could influence their future professional practice.

Generally, co‐producers indicated that the model more accurately reflected the complexity of childhood health than simpler models that solely focus on obesity (operationalised as BMI) as the outcome. While some might argue that a model which adopts a non‐stigmatising outcome and accounts for structural factors should have been the goal from the outset, it is nevertheless important to consider that many public health initiatives and policies continue to be framed around narrow constructs such as BMI. For example, the National Child Measurement Programme in England collects data on every child's height and weight in two year groups (to calculate BMI), but not on indicators such as healthy eating or mental well‐being [36].

There was a strong feeling from people with lived experience that research and services repeat the same mistakes repeatedly, embedding stigma and focusing on individuals rather than the wider context. One co‐producer, who had come into the project expecting this to be the case, was delighted to find otherwise and thrilled that their contributions were having a meaningful impact. Co‐production can foster mutual respect and create safe spaces where diverse perspectives are heard and valued (e.g., [37, 38]). Being able to articulate a perspective that challenges the heterodoxy, to have that perspective recognised, and to feel empowered in doing so is a strength of co‐production [3, 9, 39]. The experience of this co‐producer attests to this strength. However, the same co‐producer felt the final model did not go far enough in shifting the dial from stigmatising people and children in relation to weight. We considered this sense of disappointment as an example of the gap between the ideals of co‐production and the reality of its implementation [40], where reaching consensus can be challenging among co‐producers holding different experiences [17, 41]. In our case, this ‘gap’ also stemmed from a tight timeline, where additional time and space for dialogue could have fostered a clearer shared understanding of the expected outcomes and potentially greater satisfaction with the final output [41].

There was also some feedback from stakeholders in Workshop 3 that the logic model was perhaps too complex to be usable by its intended audience. The participants also noted visual elements that could be refined to make the balance of the different factors clearer (e.g., one participant noted that some of the levels had larger boxes than others, which could give a misleading impression about their relative importance).

### 4.1 Reflections From the UCL‐Based Co‐Producers

Co‐production inputs changed the focus and our collective understanding from a potentially stigmatising focus on obesity towards a more holistic understanding of childhood health. This focus also allowed for greater consideration of the social determinants of health and the broader macro‐level factors influencing children's health.

Co‐production gave us legitimacy in stepping away from academic conventions that prioritise clinical measures of health. We felt empowered being part of a co‐production team to take on a perspective that was in alignment with our knowledge of evidence in the area and our values and experiences as people. Co‐producing the model also underscored the stigmatising capacity of labels that indicate health and ill‐health.

We reflected that while the model was co‐produced in workshops, some AG members became co‐producers. We felt we had created a safe space in both the workshop and AG for interaction rather than extraction [40]. We viewed the ability to have discussions about *how* to co‐produce the model and start conversations on sensitive topics as a benefit of having a separate AG. However, in future exercises, having more crossover between advisory and co‐production groups would be beneficial to working more cohesively as a co‐production team.

In previous exercises, academic teams have occupied a neutral role in facilitating the smoothing of relations between other stakeholders (e.g., between marginalised communities and empowered others such as service providers and policymakers) (see [42]). In our case, UCL‐based co‐producers may have been initially regarded as the historically empowered stakeholder, representing institutions that had been setting the discourse on child health research. In developing the logic model, we attempted to adopt an enquiring and responsive approach to signal our willingness to work collaboratively to critically examine existing paradigms around health. However, our usual ways of working historically have not involved co‐production, and this transition was not always comfortable or easy to implement. As researchers working mainly in public health evidence synthesis, we can often be, or feel, removed from the intended beneficiaries of our research. Co‐production challenged this. For example, dealing with the disappointment of a co‐producer was uncomfortable, although it illuminated the gravity of the work. For most of the UCL‐based co‐producers, this was our first (or near‐first) experience of co‐production, and it is perhaps unsurprising that this first project represents a learning curve. We have continued to embed co‐production within our subsequent projects and to embrace the steep learning curve each time.

One limitation of our reflexive approach was that we tended to reflect at different times and using different approaches to co‐producers based outside our institution. Opportunities for whole‐group reflexivity were more limited, and reflexivity with co‐producers based outside our institution was mediated by the Co‐production Collective. This approach could be viewed as reinforcing rather than disrupting power hierarchies [43].

## 5 Discussion

Our aim here was to provide an example of the value of co‐production in developing complex theories that can be the basis of later research, in our case, evidence synthesis. We focussed our account on the shift in the health outcome that resulted from involving a diverse team in the development of the logic model, as well as the features of context that were theorised to influence children's health, which ultimately aligned with upstream public thinking [44].

Our model contains a number of features associated with intervention complexity, including its focus on theorising contextual features that may interact with intervention features [19, 45] and a high number of upstream and distal factors, with individual factors broadly treated as mediators [46]. Other forms of complex causal relationships were also represented within the model. For example, conjunctural causation was recognised with the need to ‘make healthy food more convenient AND more affordable’ within the model to improve HEPAM. Within the confines of the workshops, there was insufficient time for further theorising about other forms of complex relationships, although this would be a natural extension of the work undertaken.

### 5.1 The Strengths and Challenges of Co‐Producing a Logic Model

While public involvement is recommended in the development of logic models (see, e.g., [6, 46]), there were few examples at the time in which this study took place describing how public involvement influenced the contents of the model. Since then, a wider body of literature has emerged that documents that public involvement is possible and beneficial to the creation of theory. These studies emphasise the role of working with public contributors in strengthening understanding between researchers and stakeholders (e.g., [47, 48]), through developing a common language between contributors with different perspectives (e.g., [47, 49]) that can help to create research that represents a deeper understanding of a phenomenon that is perceived to be more relevant and applicable [15, 48, 49, 50, 51]. While previous studies identify that co‐producing research brings a transdisciplinary perspective that integrates different types of knowledge into research [9, 11, 15], this study illustrates the change this brings in practice, and the way in which co‐production can shift the focus of public health research towards a less stigmatising approach. This may further support the argument that co‐production aligns with a complex systems perspective [9]—in this case, where the outcome of the theorising emerged as a property of the co‐production process itself.

A limitation of our work is the absence of the direct voice of children. There exists a range of different approaches available for involving children and young people in research (e.g., [52, 53]), and the benefits of doing so are extensively reported—including empowerment and skill development among children and young people, enhanced insights gained, changing mindsets of other stakeholders involved in research, and ethical alignment with children's rights (e.g., [53, 54, 55, 56]). While our co‐producers with lived experience included some young people (aged 18–24), we nevertheless acknowledge that the absence of direct perspectives from children is a substantial omission. This decision was driven by:

i. a desire for the logic model to reflect multiple perspectives, including those of adults (teachers, policymakers, etc.);
ii. concern about the practicalities and risks of bringing children and adults together to discuss a sensitive topic; and
iii. pragmatic constraints related to project resourcing, which limited our ability to create an environment where children could be meaningfully involved.

A larger, better‐resourced study might have been able to, for example, increase the number of co‐production workshops to allow separate sessions for adults and children. This omission is not a weakness of co‐production as a methodology, but a reflection of the conditions under which co‐production took place (see [3, 9, 18] for further discussion around the perceived limitations of co‐production vs. failures to uphold its core principles).

Challenges and difficulties in public involvement in logic models are highlighted in the literature, including difficulties in reconciling different perspectives [17], uneven participation among group members, and a poor connection between the logic model and its intended use [50]. This latter factor is a particular risk for our example, where we intended to use the logic model to support evidence synthesis, and where there exists a risk that the richness of the model is not adequately reflected within published literature. This is not a deficiency of the logic model or the theorising per se, but a limitation of published literature, particularly the intervention literature, that we would draw upon in later evidence syntheses. Our model serves to illuminate the gap between what is theorised to influence children's health and what is measured within studies.

The model also presents a challenge around how and where to intervene. Organisations such as schools typically have little leverage in changing higher‐level factors in the system (e.g., at the socio‐political level). Nevertheless, the influence of these system‐wide factors on children's health remains important to acknowledge and theorise. This tension mirrors some concerns around the ‘cruel optimism’ of co‐production, and the extent to which co‐producers are shouldered with responsibilities to address problems that are beyond their remit to address [12, 13]. In our study, the goal of the research was always emphasised as describing and understanding systems rather than intervening. Nevertheless, as co‐producers we held diverse lived and professional experiences, which did not always align when developing the model [17]. For some, co‐production may have presented a form of ‘cruel optimism’, in terms of expectations about how the focus of the research should shift.

Some have questioned the benefits of deep co‐production in research due to concerns about time and resources [17]. We acknowledge that setting out to co‐produce a logic model was a complex undertaking, although we also reflect that co‐production is about conducting inclusive research, and working inclusively takes time. Nevertheless, in this study, more time was needed to fully establish what inclusivity meant to us as a group to avoid oversimplification and assumptions of unanimity in our discussions [57], and to use our diversity to ensure progression in our discussions from unhelpful tropes and deficit models [58]. For example, the logic model includes references to ‘cultural factors’ and ‘cultures of cooking’, but such broad concepts need further unpacking lest they be adopted as ways to blame ‘culture’ for differences in the health status of minority groups [58].

Both co‐production and the incorporation of complexity perspectives can be seen as resource‐intensive. Concerns about the resourcing of co‐produced research echo those in obesity research, particularly regarding the health benefits of continued investments in complexity‐oriented approaches [59]. This present study persists in framing obesity as a complex condition [59]. However, it also challenges the adequacy of relying solely on BMI to assess children's obesity and overall health. The National Child Measurement Programme, England's only annual child health census (with equivalents in other UK countries) [36], exemplifies this narrow focus by collecting only height and weight data, a trend common in trials and evidence syntheses. While we do not advocate abandoning BMI measures, we question why other health indicators lack similar prominence. Consequently, rather than questioning the utility of complexity‐oriented approaches, we instead argue that the complexity perspective adopted within some obesity research [60], such as whole system or whole school approaches (see [61, 62]), needs to be reflected more widely across the body of literature. The logic model suggests that both a whole system *and* whole outcome approach to conceptualising children's health may be better aligned with lived and professional experiences. We recognise that adding complexity could be viewed as a further barrier to intervention and ultimately health improvement [59] and that shifting focus from solely BMI to broader measures is difficult when services and expectations are entrenched. Nevertheless, although a full investigation is beyond the scope of this paper, a whole outcome approach may better reflect the complex decisions faced by public health policymakers, especially under budget constraints [63].

Many of the limitations around co‐production that we experienced reflect inadequate time and resources. Research systems—including funding and research ethics systems—can serve to hinder the enactment of co‐production and undermine the potential benefits. Overall, while we have provided a candid account of the challenges of co‐producing a logic model, the findings here clearly attest to the strengths that co‐production brings to theorising, as we review below.

### 5.2 The Contribution of the New Model

The logic model challenges research focussed narrowly on BMI and individual behaviours, a shift driven by public involvement and distinct from the earlier Foresight Obesity Map [26]. Others have also critiqued the Foresight map, leading to alternative systems maps. McGlashan et al. [17] developed a childhood obesity map with input from schools, health services and local organisations, highlighting missing school‐related variables and producing a map that was more closely aligned with locally relevant and feasible intervention strategies [17]. Similarly, Luna Pinzon et al. [64] created an adolescent obesity map (a causal loop diagram) using input from researchers, adolescents and stakeholders. While groups worked in parallel, adolescents' lived experiences added depth to understanding environmental interactions. Both examples retained a focus on obesity, though Luna Pinzon et al. [64] framed it through obesity‐related behaviours (namely adolescents' dietary behaviour, physical activity, sedentary behaviour and sleep).

Where our own model contributes to the literature is through sending clear signals that (i) the historical focus on childhood obesity—understood through a single clinical measure (BMI)—is stigmatising and can belie the complexity of factors that contribute to poor health in childhood (represented through the focus on HEPAM); (ii) many of the factors that are theorised to contribute to poorer health, and that influence the capacity of schools to improve child health, occur at the policy level; and (iii) theories co‐produced with a broad swathe of co‐producers emphasise the role of the social determinants of health and serve as a useful challenge to those that focus heavily on narratives of personal responsibility and individual determinism in children's health. If the model is to be redeveloped, further exploration may be useful around how forms of collective, partnered or relational agency in children's health can be theorised, which would help to advance debates beyond intractable binaries between individual/parental responsibility and broader societal responsibility for children's health.

## 6 Conclusions

Our work underscores that childhood obesity is a clinical diagnosis for a state of poor child health that has stigmatising consequences. Working as a large team with diverse experiences and perspectives challenged orthodox academic conventions that instinctively gravitate towards medicalised understandings of health, and which tend to look for individual‐level, proximal or downstream causes of poorer health. Among the UCL‐based part of the co‐production team, despite our own values and research experiences, we have reflected that without co‐production, we may have repeated these same conventional explanations, thereby continuing to embed stigma and focus on factors reflecting personal responsibility rather than the wider context.

At the time of writing, advocating for complexity perspectives, the implementation of co‐production, and the embedding of inclusivity and diversity into research appears to be at odds with public policy, particularly in the United States. However, by not integrating these elements into scientific research, we risk creating an even larger divide between scientific practice, public policy and people's lived experience. The emphasis in the model on upstream factors provides a timely reminder of the role of policy in protecting health, at a time where public health budgets have been subject to ‘death by a thousand cuts’ recently in England [65] and where public health is under attack in the United States [66].

## Author Contributions

**Dylan Kneale:** conceptualisation, data curation, formal analysis, funding acquisition, investigation, methodology, project administration, resources, supervision, visualization, writing – original draft, writing – review and editing. **Alison O'Mara‐Eves:** conceptualisation, data curation, formal analysis, funding acquisition, investigation, methodology, project administration, resources, supervision, visualization, writing – original draft, writing – review and editing. **Bridget Candy:** data curation, formal analysis, investigation, methodology, writing – original draft, writing – review and editing. **LizzieCain:** investigation, methodology, writing – review and editing. **Jessica Catchpole:** methodology, supervision, writing – review and editing. **Angela Chesworth:** methodology, supervision, writing – review and editing. **Sandy Oliver:** conceptualisation, funding acquisition, investigation, methodology, supervision, writing – original draft, writing – review and editing. **Katy Sutcliffe:** conceptualisation, funding acquisition, investigation, methodology, supervision, writing – original draft, writing – review and editing. **Niccola Hutchinson Pascal:** funding acquisition, investigation, methodology, project administration, resources, supervision, validation, writing – review and editing. **James Thomas:** conceptualisation, funding acquisition, investigation, methodology, supervision, writing – review and editing.

## Conflicts of Interest

The authors declare no conflicts of interest.

## Data Availability

The data that support the findings of this study are available in the main body or are freely available here: https://sites.google.com/view/cephi-project/logic-model. No other data were collected or are available due to privacy or ethical restrictions.
